# Machine learning-predicted chromatin organization landscape across pediatric tumors

**DOI:** 10.1038/s41598-026-44925-3

**Published:** 2026-03-28

**Authors:** Ketrin Gjoni, Shu Zhang, Rachel E. Yan, Bo Zhang, Daniel Miller, Jeffrey P. Greenfield, Adam Resnick, Nadia Dahmane, Katherine S. Pollard

**Affiliations:** 1 Gladstone Institute of Data Science and Biotechnology, San Francisco, CA 94158 USA; 2 Department of Epidemiology and Biostatistics, University of California, San Francisco, CA 94158 USA; 3 Department of Neurological Surgery, Weill Cornell Medicine, New York, NY 10065 USA; 4 Center for Data-Driven Discovery in Biomedicine, Children’s Hospital of Philadelphia, Philadelphia, PA 19104 USA; 5 Bakar Computational Health Sciences Institute, University of California, San Francisco, CA 94143 USA; 6 Chan Zuckerberg Biohub, San Francisco, CA 94158 USA

**Keywords:** Cancer genomics, Computational models, Machine learning, Epigenomics, Structural variation

## Abstract

Structural variants (SVs) are increasingly recognized as important contributors to oncogenesis through their effects on 3D genome folding. Recent advances in whole-genome sequencing have enabled large-scale profiling of SVs across diverse tumors, yet experimental characterization of their individual impact on genome folding remains infeasible. Here, we leveraged a convolutional neural network, Akita, to predict disruptions in genome folding caused by somatic SVs identified in 61 tumor types from the Children’s Brain Tumor Network dataset. Our analysis reveals significant variability in SV-induced disruptions across tumor types, with the most disruptive SVs coming from lymphomas and sarcomas, metastatic tumors, and germline cell tumors. Dimensionality reduction of disruption scores identified five recurrently disrupted regions enriched for high-impact SVs across multiple tumors. Some of these regions are highly disrupted despite not being highly mutated, and harbor tumor-associated genes and transcriptional regulators. To further interpret the functional relevance of high-scoring SVs, we integrated epigenetic data and developed a modified Activity-by-Contact scoring approach to prioritize SVs with disrupted genome contacts at active enhancers. This method highlighted highly disruptive SVs near key oncogenes, as well as novel candidate loci potentially implicated in tumorigenesis. These findings highlight the utility of machine learning for identifying novel SVs, loci, and genetic mechanisms contributing to pediatric cancers. This framework provides a foundation for future studies linking SV-driven regulatory changes to cancer pathogenesis.

**Supplementary Information:**

The online version contains supplementary material available at 10.1038/s41598-026-44925-3.

## Introduction

Pediatric brain tumors (PBTs) are the most common solid cancers in children and have seen little improvement in treatment or understanding compared to other childhood cancers^1^. One reason is that the genetic causes behind many PBTs remain unclear, and efforts are ongoing to dissect the underlying molecular mechanisms that promote tumorigenesis in these cancers. Structural variants (SVs)–large deletions, duplications, inversions, insertions, and chromosomal rearrangements of genomic regions–are major drivers of cancer that play an important role in shaping the transcriptomic landscape of PBTs^2^. SVs can disrupt tumor suppressors or amplify oncogenes by fusing genes, altering regulatory elements, or directly disrupting the genes themselves. In cancer, SVs have been shown to create harmful gene fusions such as *BCR-ABL* in leukemias and *MYB-QKI* rearrangements in pediatric gliomas^3,4^. Overall, PBTs have higher rates of SVs compared to other pediatric tumors^5^ and their SV patterns differ from those observed in adult tumors. Recent studies found both simple and complex SV signatures in PBTs, associated with differences in replication timing, GC content, and breakpoint proximity to repetitive elements, telomeres, and topologically associating domain (TAD) boundaries^6^.

More recently, SVs have been suggested to cause cancer through alterations to genome organization^7^. In humans, the genome is folded into higher order structures, ranging from small loops of only a few kilobases (kb) in length to larger TADs that span multiple megabases (Mb) of DNA^8^. By measuring genome organization through conformation capture experiments, the effect of SVs on structures like TADs has been implicated in various PBTs. The deletion of a TAD boundary can merge two TADs and cause genes from one TAD to hijack enhancers from the previously distinct other TAD and become overexpressed. Enhancer hijacking has been shown to lead to the activation of oncogenes across cancer types^9,10^, including pediatric high-grade gliomas^11^ and medulloblastomas^12^. In ependymomas, the SV-caused formation of neo-TADs has been shown to affect essential genes^13^. SVs that result in rearrangements of genomic contacts have been shown to upregulate *TERT* and contribute to neuroblastoma^14,15^. These studies clearly highlight the contribution of SVs to cancer progression through altered genome structure.

We hypothesize that SV-mediated disrupted genome organization contributes to PBTs at large, beyond these individual anecdotal examples. The Children’s Brain Tumor Network (CBTN)–a collaborative and international effort collecting a rich database of pediatric tumor multi-omics and clinical data^16^–allows us to comprehensively study PBT SVs. However, testing the effect of hundreds of thousands of SVs experimentally would be costly, time-consuming, and impossible for larger SVs. To overcome this, we turn to machine learning (ML) to predict 3D genome folding patterns in high throughput and at low cost. The Akita model predicts chromatin contact maps from a ~ 1 Mb DNA sequence with high accuracy^17^. This sequence-based model enables the use of in silico mutagenesis (ISM) to quantify the isolated effect of individual SVs. SuPreMo-Akita streamlines ISM with Akita by making predictions for DNA sequences with and without an SV of interest and measuring the effect of each SV on the surrounding chromatin contacts^18^. With these tools, we are equipped to systematically test millions of SVs in silico, and prioritize ones predicted to be most harmful.

Here, we applied SuPreMo-Akita ^18^ to nearly 300,000 SVs from pediatric cancer patients in the CBTN cohort to evaluate SV disruption to genome folding and find patterns across and within individual tumor types. We investigated regions that experienced recurrent disruptions to genome organization across tumor types, evaluated variant effects on putative enhancers, and prioritized candidate variants that disrupt tumor-associated genes.

## Results

### Overview of SV profiles across CBTN tumors

**Fig. 1:**
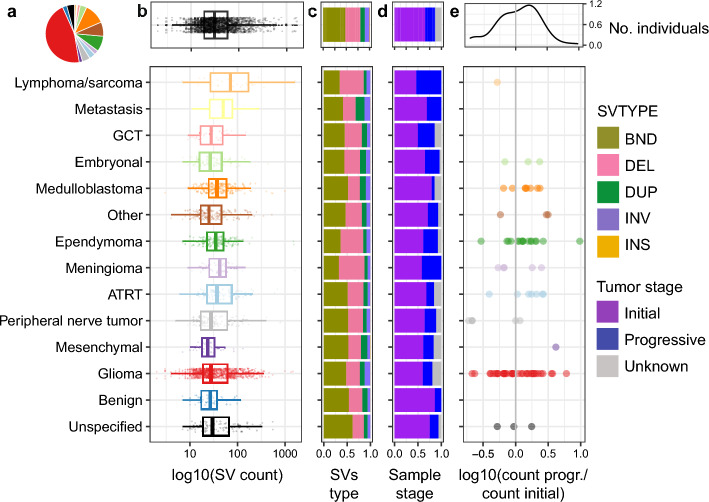
Summary of SV profiles across 14 pediatric tumor categories. (**a**) Distribution of the number of tumor samples per broad tumor category. (**b**) Distribution of variant lengths across tumor categories. Each point represents a variant; multiple samples and tumor types are included in each category. (**c**) Fraction of SV types across tumor categories, calculated from all SVs in all samples in each category. (**d**) Fraction of tumor stages across samples, calculated from all samples in each category. (**e**) Log fold change in number of variants in progressive over initial samples from each participant. Only participants with both sample types are included. For participants with multiple initial or progressive samples, one of each was randomly selected. Only variants that were scored are shown here, although these trends hold when looking at all CBTN variants. GCT: germ cell tumor, ATRT: Atypical Teratoid Rhabdoid Tumor.

We analyzed pediatric brain tumors from the CBTN cohort, where somatic SVs were called on tumor tissues from 1,843 individuals, with a median age of 9.4 years. Patients were diagnosed with 61 distinct cancer types, including some with combinations of multiple types^16^, which were grouped into 14 broader categories (Fig. 1, Supplementary Fig. 1, Supplementary Table 1, Methods). The majority of samples were gliomas (Fig. 1a), which included brainstem glioma, low- and high-grade astrocytoma, ganglioglioma, dysembryoplastic neuroepithelial tumor (DNET), gliosis, glial-neuronal tumor, gliomatosis cerebri, subependymal giant cell astrocytoma (SEGA), and oligodendroglioma (Supplementary Table 1). Across these, low grade astrocytomas were most prevalent, with 553 samples (Supplementary Fig. 1a). As seen with single nucleotide variant (SNV) mutation rates^19,20^, SV mutation rates also varied across tumor categories. On average, samples had a median of 35 SVs, with lymphomas and sarcomas having the highest SV mutation rates at a median of 71.5 SVs per sample, and mesenchymal tumors, such as Ewing sarcoma and hemangioblastoma, the lowest at 24 SVs per sample (Fig. 1b).

The dataset included SVs of five different types: deletions (DEL), duplications (DUP), inversions (INV), insertions (INS), and chromosomal translocations (BND). Overall, BNDs were the most frequent in the dataset (48%), with DELs second (33%) (Fig. 1c and Supplementary Fig. 1b). The distribution of SV types varied across tumor categories. Meningiomas had the highest proportion of DELs (55%) and lowest proportion of BNDs (33%), while metastatic tumors had the highest proportion of INVs and DUPs at 12% and 19%, respectively. Samples were obtained from both initial and progressive tumors, with around 75% from initial and 25% from progressive cases (Fig. 1d and Supplementary Fig. 1c). As expected, progressive or recurrent tumors exhibited a 1.2 times higher SV burden than initial tumors, with 56% of participants having more SVs in progressive samples (Fig. 1e and Supplementary Fig. 1d). Interestingly, in some tumor types such as peripheral nerve tumors, the opposite was true: recurrent tumors had a lower tumor SV burden in 5 of 6 participants, potentially reflecting lower heterogeneity in the progressive samples after various selection pressures.

### ML predicts disruptions to 3D genome folding resulting from somatic SVs

**Fig. 2:**
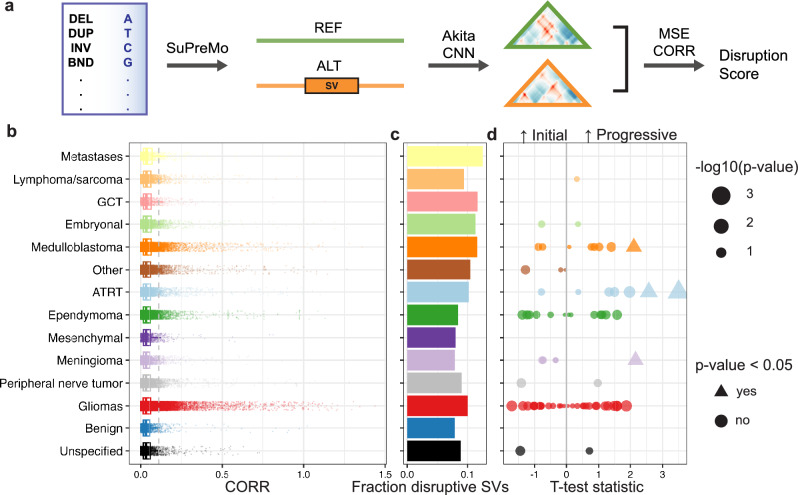
Comparison of 3D genome folding disruption scores. (**a**) Schematic for scoring CBTN SVs with SuPreMo-Akita, which takes in variants and outputs disruption scores. For each variant, length-matched reference and mutated alternate sequences are generated. Sequence pairs are inputted into the Akita model to produce contact frequency maps. The maps are compared using MSE and CORR to quantify the disruption each variant causes to surrounding genomic contacts. (**b**) Distribution of disruption scores (CORR) across tumor categories, where each datapoint is a variant. (**c**) Proportion of variants that score above the 90th percentile of all CBTN variant scores. (**d**) Statistics and *p*-values from t-tests between variant scores from progressive and initial sample pairs (Methods). A positive statistic corresponds with progressive SVs being more disruptive than initial SVs. Significant tests (*p*-value < 0.05) are portrayed using triangles while the rest are portrayed as circles.

Variants from all tumor samples were scored for their effect on surrounding 3D genome contacts using the SuPreMo-Akita pipeline^18^ (Fig. 2a, Supplementary Table 2, Methods). In brief, each variant was individually mutated into the reference genome sequence and contact frequency maps for the surrounding 1 Mb region were predicted using Akita. We compared maps corresponding to the reference (REF) and mutated (ALT) genome sequence to measure disruption of genomic contacts using mean squared error (MSE) or 1—correlation (CORR). A higher MSE or CORR score indicates that a variant is predicted to disrupt genome organization to a greater degree. While we calculate and provide both scores, in some analyses we only focus on one of them for simplicity. Additionally, while we scored all SVs available in the CBTN callset, we only showed scores for SVs that passed an additional filter for potential artifacts (Methods).

We compared CORR disruption scores across tumor categories using the median score across all variants in each sample. We found that metastatic tumors had the highest median scores across categories (Fig. 2b,c), with the highest proportion of disruptive variants at 12.5% (Fig. 2b,c). This could be because metastasized tumors have accumulated or contained damaging variants that evaded initial treatment. Lymphomas and sarcomas were the second highest scoring category, with rhabdomyosarcomas driving this trend (Supplementary Fig. 2a). Interestingly, benign tumors had the lowest median scores, suggesting that they have higher rates of passenger mutations. Neuroblastomas (in the embryonal category) and medulloblastomas, both of which have been shown to be associated with genome structure disruptions^12,14,15^, were in the top 5 highest scoring tumor categories (Fig. 2b). Most of these trends held up when using MSE disruption scores (Supplementary Fig. 3a-c).

We then looked more closely at how tumor types ranked within these categories. For some categories, like lymphoma/sarcomas, all the tumor types scored consistently high, in line with the overall category ranking. For others, like gliomas and embryonal tumors, there was a notable variation in disruption that often aligned with how aggressive versus benign the tumor types are. Within the glioma category, brainstem gliomas, gliomatosis cerebri, and oligodendrogliomas scored the highest, whereas gangliogliomas and low grade astrocytomas–both of which are considered to be less aggressive–scored low (Fig. Supplementary Fig. 2a-b). Among embryonal tumors, embryonal tumors with multilayered rosettes (ETMR), which are highly aggressive^21^, scored the highest and DNET^22^, which are considered more benign, scored the lowest. Variant effects on genome structure can be visualized in the Akita-predicted contact frequency maps, through the difference between the reference map and the alternate map with the SV and through the disruption track along the map (Supplementary Fig. 2d).

While we found that progressive tumor samples had more SVs on average than initial ones, we wanted to evaluate if they also had more damaging SVs. Overall, progressive tumor SVs score significantly higher than initial tumor SVs, with a mean of 0.053 and 0.050, respectively (Welch t-test, p-value = 2.26e-06). For a more direct evaluation, we compared SVs from an initial sample to SVs that arose in a progressive sample from the same individual, which was available for 121/1,843 individuals in the dataset (Supplementary Table 3). Overall, progressive samples had higher mean scores than initial samples (Fig. 2d), with a mean t-test statistic of 0.1 across all individuals. This difference was significant for 3/7 ATRT participants, 1/2 meningioma participants, and 1/38 glioma participants (Fig. 2d and Supplementary Fig. 2c). Higher scores in progressive samples could suggest that they are more aggressive than the initial version of the tumor.

To better understand what makes an SV disruptive to genome structure, we looked at how scores are associated with variant length and location. We found a significant positive association between disruption scores and variant length (Supplementary Fig. 2e), as seen for SVs from other disease cohorts^23^. One reason for this might be that larger SVs are more likely to encompass more sequence features that determine genome organization. To account for this, we separated analyses by SV length, when relevant. We also found that there is a relationship between whether the SV overlaps genes or not and how disruptive it is (Supplementary Fig. 2f.). For non-BND SVs, large coding SVs are more disruptive than large intronic or intergenic SVs. For BNDs–which are all noncoding–intronic SVs are more disruptive than intergenic ones.

### SuPreMo-Akita scores unveil recurrently disrupted genomic regions across tumor types

**Fig. 3:**
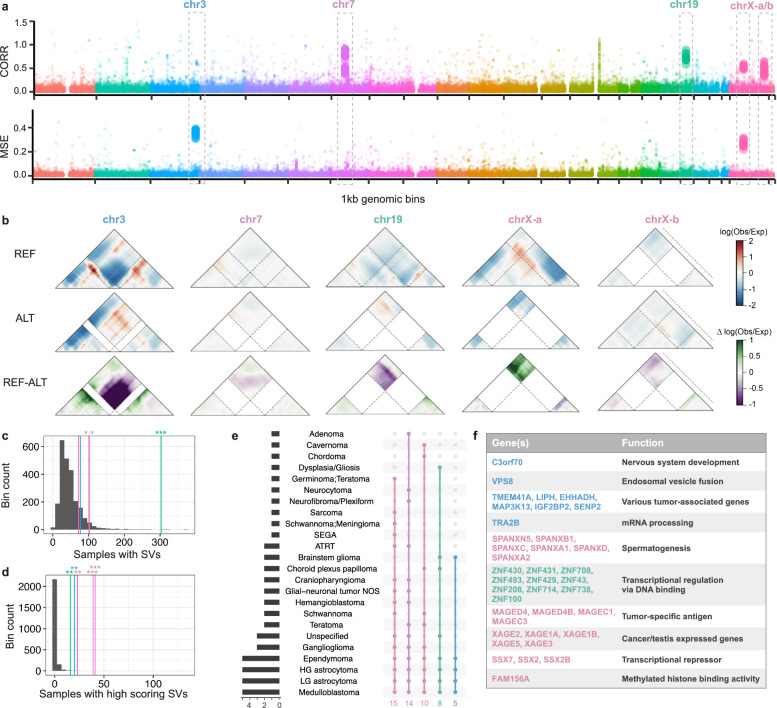
Recurrently disrupted genomic regions. (**a**) Disruption scores for the most disruptive SV for each tumor sample across 1 kb genomic bins, ordered by genomic coordinates and colored by chromosome. Sample groups were identified by UMAP on scores for the most disruptive SV (Methods). All the clusters had disruptive variants (peaks in the Manhattan plot) in the same bin, which are denoted by larger dots. Boxed peaks represent bins that are recurrently disrupted across samples and tumor types using CORR and/or MSE. (**b**) Contact frequency maps for example variants from each highlighted bin in a. Reference and alternate maps and the difference between them are shown top to bottom, and dashed lines represent the start and end of the variant. Coordinates for the windows shown, from left to right: chr3:185,492,711–186,410,215, chr7:51,757,685–52,675,189, chr19:20,879,532–21,797,036, chrX:52,172,467–53,089,971, chrX:140,948,793–141,523,916. (**c**) Distribution of the number of samples that have an SV at each one of 5 bins. Colored lines represent the number of samples that have an SV in each of the bins highlighted in a. Stars represent significance based on p-value. (**d**) Same as c but instead looking at the number of samples with an SV that passes the disruption cutoff used in a, for each bin. (**e**) Tumor types with variants above disruption cutoff in each of the 5 bins from a, and the overlap across the 5 bins. (**f**) Genes in bins from a, grouped by their function.

We next sought to look for patterns in disruption scores along the genome across tumor samples. We evaluated each 1 Mb bin of the genome based on the highest scoring variant for each sample and performed dimensionality reduction. This unveiled groups of samples that clustered separately from the rest due to having disruptive SVs in the same bin. We evaluated these bins and removed low quality predictions (Methods). This resulted in five 1 Mb recurrently disrupted regions (RDRs), where clusters of samples had independent somatic SVs that overlapped the region and scored high (Fig. 3a). All disruptive SVs in RDRs were DELs and DUPs; some samples had similar SVs overlapping the same RDR while others had a variety of variant lengths and types (Supplementary Table 4).

Changes to surrounding genomic contacts included loss of a TAD boundary in three of the RDRs (chr3, chr7, and chr19) and loss of a loop in another one (chrX-a) (Fig. 3b). While only three of the RDRs are mutated in significantly more samples than the rest of the genome (Fig. 3c), all 5 are disrupted in significantly more samples than the rest of the genome (Fig. 3d), highlighting the benefit of disruption scores in finding these regions. RDRs are disrupted in up to 5–15 different tumor types, with medulloblastoma, low- and high-grade astrocytoma, and ependymoma having disruptive variants in all 5 RDRs (Fig. 3e). Interestingly, RDRs include genes involved in nervous system development, transcription, as well as tumor-associated genes, suggesting potential involvement in PBTs (Fig. 3f).

To evaluate what makes RDRs stand apart from the rest of the genome, we looked at SV calling confidence and presence of repetitive elements (REs) at SV boundaries. SVs in all RDRs have larger confidence intervals (CIs) than DELs and DUPs in non-RDR bins, suggesting that the exact start and end SV coordinates might be imprecise (Supplementary Fig. 4a). Most RDRs have a smaller proportion of SVs with matching REs at their boundaries than the rest of the 1 Mb genomic bins (Supplementary Fig. 4b). These mostly include SINE, LINE, simple repeat and LTR elements (Supplementary Fig. 4c). The chr19 RDR, on the other hand, has a higher proportion of SVs with matching REs, which might explain why it is recurrently mutated in more samples than other bins (Fig. 3c). Overall, these findings lead to the hypothesis that variants in RDRs might play a role in tumorigenesis through disruption of genomic contacts and changes in expression of cancer-causing genes.

### Defining highly disruptive variants

While looking at broad patterns of scores helps us compare tumor types, we expect that only a small subset of variants alters genomic contacts in ways that affect transcription. To help find these variants, we applied a stringent disruption cutoff above which variants were predicted to have strong structural changes (Methods). This resulted in 363 variants above this cutoff, which we defined as “highly disruptive”, and they were present in only 34 of the 61 of the tumor types. While most high scoring SVs in RDRs described above also fell into the “highly disruptive” classification, we wanted to more broadly evaluate all highly disrupted variants and the genes they might affect. We then defined highly disrupted genes as protein coding genes within 300 kb of highly disruptive SVs that do not overlap their exons. This resulted in 2,012 genes, 249 of which were predicted to be disrupted in more than one tumor type, mostly including medulloblastoma and high-grade astrocytoma (Supplementary Fig. 5a-b). These recurrently highly disrupted genes were enriched for myosin complex and axon initial segment (Supplementary Fig. 5c).

To focus on variants that largely have an effect through disruption of genome structure, we isolated noncoding variants that passed our strongest disruption cutoff. Out of these 13 variants, 6 resulted in strong changes of contacts of relevant genes (Supplementary Fig. 6). For example, a 127,129 base pair (bp) DEL in chromosome 1 removed a TAD boundary and caused gained contacts between the TADs it was insulating, which included *AKT3*, involved in cell proliferation, differentiation, apoptosis, and tumorigenesis and *ZBTB18*, a transcriptional repressor in neuronal development. On chromosome 2, two variants create partial DUPs of TADs that contain *MYCN*–a known tumor associated gene–and *EGLN3*–implicated in renal cell carcinoma^24^–and have the potential to affect their regulation. We hypothesize that these noncoding variants, which do not directly affect genes, might impact the expression of relevant nearby genes by disrupting their distal interactions.

### Disruption is linked to regulatory regions

**Fig. 4:**
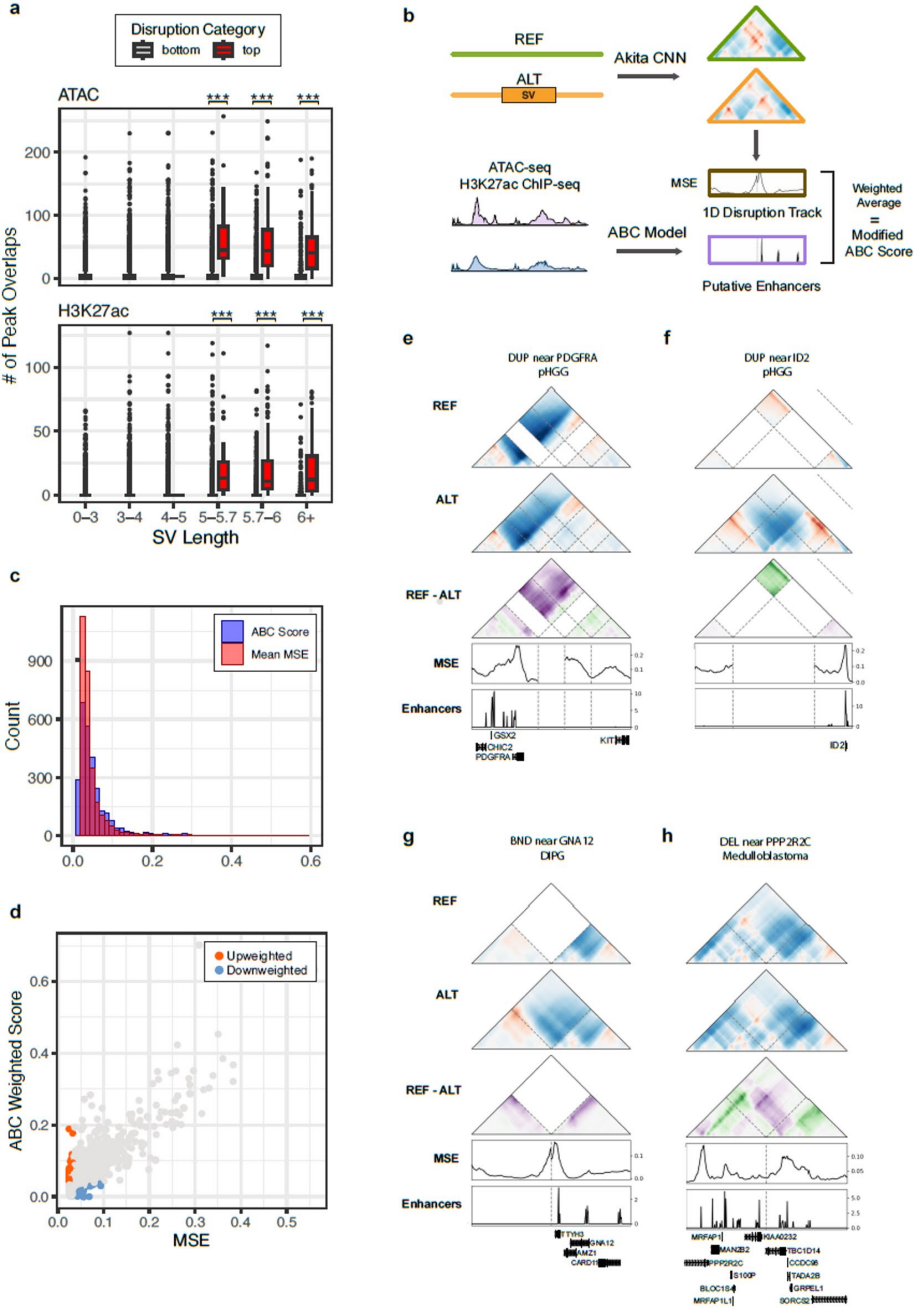
Upscaling disruption at cancer-relevant regions prioritizes previously missed SVs. (**a**) Overlap of pHGG SVs with H3K27ac peaks and accessible regions from KNS42, a pediatric glioblastoma cell line. Significant differences in overlap between disruptive and non-disruptive variants within each SV length bin are starred (Mann–Whitney U test, p < 0.0001). (**b**) Schema to calculate the ABC disruption score, which weights the contribution of predicted 3D genome disruption from an SV based on the enhancer activity in the prediction window. (**c**) Histogram of MSE and ABC disruption scores calculated for the top 10% of originally scored pHGG variants. (**d**) Scatterplot of MSE versus ABC-weighted scores in pHGG (Pearson *R* = 0.8433). Upweighted and down weighted variants are colored in orange and blue, respectively. (**e**) Example contact maps of ABC-score prioritized variants near implicated glioma driver genes *PDGFRA* (DUP at chr4:54,365,518–54,518,881) and (**f**) *ID2* (DUP at chr2:8,498,372–8,972,628) in pHGG. (**g**) A BND near *GNA12* in DIPG (BND at chr12:115,865,475–chr7:2,645,728) that was prioritized by ABC disruption scoring. The left and right regions of the horizontal axis for these contact maps depict the 500 Mb region by the breakpoints of the two translocated chromosomes. (**h**) ABC-score prioritized DEL near *PPP2RC* in medulloblastoma (DEL at chr4:6,948,839–6,949,029).

Chromatin alterations with functional consequences may contribute more directly to cancer progression, so we focused our analyses on regulatory regions across the genome. To evaluate variant effects on regulatory elements, we identified H3K27ac ChIP-seq peaks and accessible chromatin regions from tumor type-matched cell lines as indicators of potential enhancer activity. We first examined the association between these regions and variant disruption across tumor types. For pediatric high-grade gliomas (pHGGs), we found that highly disruptive variants were more enriched in H3K27ac peaks and ATAC-seq peaks when compared to the rest of the scored variants, even after controlling for SV length (Methods, Fig. 4a). This trend also held across an additional three tumor types for which we had the corresponding epigenetic data: atypical teratoid rhabdoid tumors (ATRT), diffuse intrinsic pons glioma (DIPG), and medulloblastomas (Supplementary Fig. 7a-c). This suggests that there is an interplay between regulatory element contacts and disruptive variants. Motivated by this finding, we extended our SuPreMo-Akita scores to quantify variant effects on regulatory elements and to prioritize those variants most likely to disrupt enhancer-promoter contacts.

To upweight variants found at regulatory regions, we took inspiration from the Activity-By-Contact (ABC) Model ^25^, which predicts the strength of enhancer-gene interactions based on information from experimental ChIP-seq, ATAC-seq or DNase-seq, and Hi-C. Using putative enhancers calculated by the model, an “ABC disruption score” for variants from four of the tumor types was determined. In brief, for any loci containing a putative enhancer, the ABC activity score was multiplied by the genome folding disruption score for that region. The final ABC disruption score is a weighted average of the ABC activity score and the 1D disruption score across the 1 Mb region surrounding the variant (Fig. 4b, Methods). We applied this scoring metric to the top 10% of disruptive variants across pHGGs, DIPGs, ATRTs, and medulloblastomas (Supplementary Table 5).

We found that the ABC weighted scores were similar in distribution to and highly correlated with the original disruption scores (Fig. 4c,d), suggesting that patterns of genome folding disruption are not skewed towards regulatory regions for most variants. However, we expect that causal variants will drive disruption specifically in functionally important regions, such as enhancers, promoters, and insulating TAD boundaries. In order to identify such variants, we looked at SVs that increased in disruption with the ABC scoring approach compared to the original MSE score (Supplementary Fig. 8a, Methods). In turn, these upweighted SVs cause focused disruption at enhancer contacts.

The proportion of SV types was consistent across up- and down- weighted SVs, while scores for INVs were largely not changed, likely because of their low scores. There was a trend with SV length: larger SVs were more likely to be down weighted given the smaller remaining region in the prediction map that was evaluated (Supplementary Fig. 8b-c). Genes overlapping upweighted variants were found to be involved with the endoplasmic reticulum and immune related gene ontology categories. (Supplementary Fig. 8d, Supplementary Table 6). These upweighted variants also affected known cancer genes and genes implicated in cell cycle processes. For pHGG, one of the top three upweighted variants involved *PDGFRA*, a gene encoding a receptor tyrosine kinase that regulates proliferation and survival, and one frequently mutated in gliomas^26^. We saw both coding and noncoding pHGG variants near *PDGFRA* that disrupted chromatin contacts, including a DUP that increased predicted genomic contact for an enhancer at the start of the *PDGFRA* gene (Fig. 4e). A different DUP drastically decreased genomic contact at regulatory elements near *ID2*, a transcriptional repressor that regulates cell differentiation and tumor proliferation in pHGG and other cancers (Fig. 4f)^27,28^. Furthermore, enhancers in both *PDGFRA* and *ID2* have previously been shown to be targets of recurrent SVs in pHGG^29^, suggesting that the ABC disruption scoring approach identified variants with functionally relevant regulatory roles.

These trends were consistent across other tumor types. Additional upweighted variants included a DEL in a DIPG sample that created a gain of contact upstream of *GNA12*, which has been shown to be upregulated in gliomas (Fig. 4g)^30^. *GNA12* activates the RhoA/ROCK signaling pathway, which is involved in cancer cell growth and progression. A DEL in medulloblastoma caused a predicted loss of contact at an enhancer near *PPP2R2C*, a gene involved in synaptic plasticity that has been linked to brain cancers and other mental disorders (Fig. 4h) ^31^. Overall, the weighted scoring approach emphasized changes at active enhancer regions and identified variants that were overlooked with the overall disruption score.

### Upweighted variants in ATRT disrupt candidate cancer genes

We placed a greater focus on ATRT, given that it had the highest fraction of high-scoring variants, and highest scoring initial tumor variants. Additionally, ATRT had a slightly larger number of variants with an increase in rank after ABC scoring (Supplementary Fig. 7d). ATRT is primarily driven by loss of *SMARCB1*, a subunit of the SWI/SNF chromatin remodeling complex. However, clinical heterogeneity in ATRT is unexplained by the loss of *SMARCB1* alone ^32^, and efforts are ongoing to understand the remaining genetic factors that may contribute to this deadly tumor. To identify possible candidate genes that contribute to ATRT tumorigenesis, we first looked at the most upweighted variants. Several of these variants occurred near genes linked to chromatin organization and *SMARCB1*, such as *CCND1* and *BCL7C* (Supplementary Fig. 9a-b). Studies have shown that *CCND1* is a target of 3D genome changes in cancer, and that loss of *SMARCB1* induces *CCND1* deficiency ^33,34^. *CCND1* codes for a component of the CDK complex, which in turn regulates the cell cycle and cell growth. Like *SMARCB1, BCL7C* is a subunit of the SWI/SNF complex, and it has been suggested as a biomarker in gliomas ^35^.

**Fig. 5:**
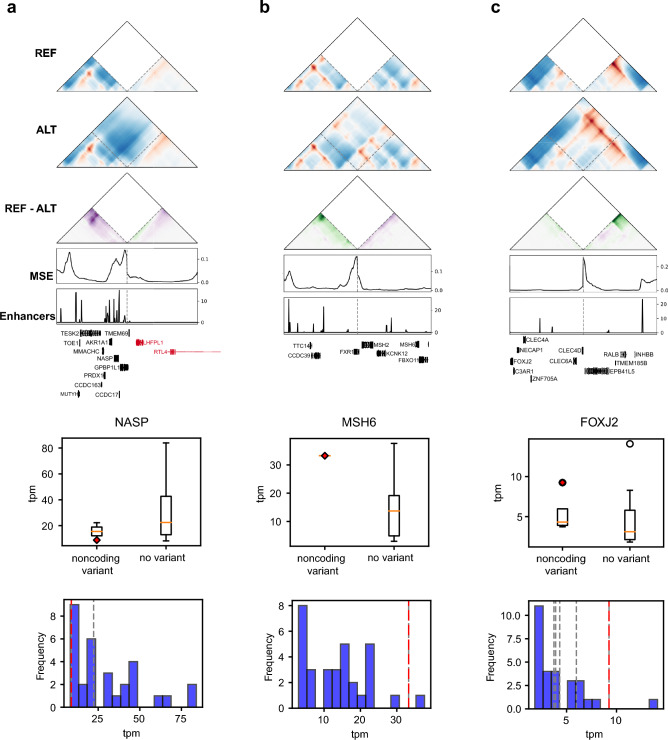
Upscaling disruption at cancer-relevant regions suggests potential new disruptive variants in ATRT. (**a**–**c**) Candidate BND variants upweighted by the ABC disruption score. Disruption is high at putative enhancers near (**a**) NASP (chr1:45,703,450–chrX:112,696,292), (**b**) MSH6 (chr3:180,939,147–chr2: 47,413,958) and (**c**) FOXJ2 (chr12:8,521,326–chr2:120,042,934). For each panel, the middle boxplots show gene expression (tpm) for samples containing a noncoding variant versus no variant at all near the gene of interest. The red diamond marks the sample containing the variant shown at the top. The bottom histograms show the distributions of gene tpm values in samples with no variants near the gene. Dotted lines indicate the expression values for samples with noncoding variants near the gene. The sample containing the example variant is marked with a red dashed line.

We also highlight a few upweighted variants with corresponding expression changes at nearby genes. All of these variants were BNDs, with high genome folding disruption at putative enhancers of the translocated genes (Fig. 5a–c), consistent with enhancer hijacking. These include *NASP*, *MSH6*, and *FOXJ2*. When comparing the samples containing these variants with other ATRT samples containing no variants near these genes, the variant-containing samples had gene expression values more extreme than other samples. The samples with variants near *NASP*, *MSH6*, and *FOXJ2* had expression lower than 31/32 samples, higher than 31/32 samples, and higher than 27/28 samples, respectively. Each of these genes have been implicated in other cancers such as glioblastomas, melanomas, ovarian, and colorectal cancers, and they help regulate processes including histone transport, neural development, DNA damage repair, and cell growth^36–38^. We observed similar patterns for several of the other genes near these variants (Supplementary Fig. 10), supporting our hypothesis that the SV-driven genome folding alterations may be closely tied to functional effects. Overall, these results demonstrate that ML models can prioritize SVs that disrupt 3D genome folding near neurological and cancer-relevant candidate genes, which help generate new hypotheses regarding their functional roles.

## Discussion

Here, we present an ML approach to rapidly and systematically screen an unprecedented number of variants to test if they alter 3D genome organization. We applied this computational approach to characterize somatic SVs across 61 different tumor types from the CBTN cohort. Our data revealed that different tumor types experienced a range of genome folding disruption, with larger SVs and SVs from progressive samples being more disruptive. We found regions that were recurrently disrupted in up to 15 tumor types, which contained genes linked to tumorigenesis and neural system development. Strikingly, several of these 3D disruption hotspots were not mutation hotspots, indicating that SVs in these loci have an elevated propensity to alter genome folding and that our method was essential for identifying their convergent effects. Additionally, we demonstrated that a small number of highly disruptive SVs near cancer genes can be prioritized from a pool of hundreds of thousands of variants, most of which have minimal predicted effects on genome folding. Prioritized SVs were associated with active regulatory regions, leading us to integrate epigenetic data into a new ABC disruption scoring approach to prioritize variants relevant in cancer. Focusing on four tumor types, we identified candidate causal SVs that may contribute to tumorigenesis due to changes in genome structure affecting genes with known and plausible roles in cancer.

Our approach has several limitations, beginning with the SV set itself. SVs are often complex and co-occur, making it challenging to determine precise breakpoints or the sequence of individual SV events, particularly with short-read sequencing. As a result, SV detection involves a balance between calling false positives and false negatives. For example, homologous or repetitive regions can lead to misaligned reads and false-positive SV calls, whereas low tumor purity or high tumor heterogeneity can reduce the variant allele fraction of a true SV below the detection threshold, producing false negatives. Because tumor purity varied across samples and sequencing coverage data was unavailable for our SV cohort, SV detection sensitivity was likely non-uniform across samples, potentially biasing comparisons of SV burden and disruption. Additional factors, such as read support at breakpoints, overall read depth, and alignment quality, also influence SV calling^39,40^. Benchmarking studies have shown that certain callers perform better across SV classes, and Manta, which we use here, demonstrates high precision and sensitivity ^41^. Emerging long-read sequencing technologies can help resolve ambiguous calls, but they have not yet been widely applied in cohort-scale studies ^40^.

In addition, our screen is dependent on the Akita algorithm and its learned sequence determinants of genome folding. Akita can only make predictions for a 1 Mb window, preventing us from evaluating larger SVs or broader structural changes (e.g. compartment changes). Additionally, Akita was not trained on the specific cell types relevant to pediatric tumors. This is somewhat mitigated by the fact that genome organization is relatively stable across tissues and by our use of epigenetic data from cancer cell lines to upweight chromatin interactions at enhancers in relevant tumor types. Previous studies using Akita have found that predictions in one cell-type were experimentally validated in another cell type, giving us high confidence in Akita’s predictions ^23^. Nonetheless, we recognize that some context-specific chromatin interactions may be missed. To gain further confidence and insights into our results prior to experimentation, they can be combined with predictions from other ML models, such as those predicting chromatin accessibility or transcription factor binding^42^. Despite these limitations, our predictions serve to generate new, testable hypotheses about hotspot loci and candidate causal variants that can be evaluated in future laboratory studies. Experimental validation will be a critical next step.

WGS is increasingly used in the clinic, and SuPreMo-Akita can contribute to the interpretation of variants beyond obvious gene fusion and inactivation events. The availability of variant data for larger cohorts will necessitate the use of computational strategies, such as ours, to narrow the search space in research projects aimed at elucidating causal mechanisms and identifying new pathways that can be targeted to treat or prevent cancers. This approach serves as a framework that can be generalized to other cancers, as the underlying mechanisms of genome-folding mediated gene regulation is maintained across different tissue and disease contexts. By applying similar ML-based strategies to different cancer datasets, researchers can systematically identify functional SVs that may drive tumorigenesis. Finally, while clustered rearrangements such as chromothripsis likely cause more drastic changes to genome organization and analyzing haplotype-scale effects represents an exciting future direction, our one-by-one approach allows us to isolate and interpret the impact of each SV individually. Both perspectives are important, and we emphasize here the value of characterizing single-SV effects as a foundation. Overall, this study shows the utility of ML in identifying functional SVs, and further strengthens previous evidence that SVs contribute to tumorigenesis through changes in genome folding.

## Methods

### Human tissues

All data used in this study is publicly available and was previously published by the CBTN^16^. We did not perform experiments on or otherwise use human tissue samples.

### Data used

SVs were previously generated by the CBTN^16^ with the Manta SV caller (v1.4.0)^43^. Somatic SVs were called, and only those that passed filtering (‘PASS’) were used. Gene expression data was previously measured and available on the CBTN database in the form of tpm. Variants were previously annotated using AnnotSV^44^. Annotated data was missing for 2/2,386 due to an issue with the vcf file. We grouped tumor types into tumor categories based on their cell types of origin. We analyzed all tumor types available within the CBTN study, which included tumors located in any part of the CNS. Tumors like lymphomas, which can be found in other regions of the body, were only included in this study if they were in CNS regions. Similarly, CNS tumors that metastasized from primary tumors elsewhere in the body were also included. CBTN previously annotated tumors as “initial” versus “progressive”, and we used their definitions.

### Scoring variants with SuPreMo

SuPreMo-Akita^18^ was used to score all CBTN somatic SVs for the disruption they cause to genomic contacts in the surrounding ~ 1 Mb region. We report augmented scores, which represent the median or mean score for the following input DNA sequences: no augmentation, − 1 bp sequence shift, + 1 bp sequence shift, and reverse complement of the sequence. MSE and CORR were used to score the differences between the reference and alternate contact frequency maps, with higher scores corresponding to more disruptive variants. Discussion of trends was focused on CORR scores but most trends were consistent when using MSE (Supplementary Fig. 3). Scores only reflect changes to contacts in the map outside of the SV, which is masked from the heatmap. Due to Akita’s sequence length limitation, variants greater than 500 kb in length were removed from the analyses since the remaining portion of the map was deemed insufficient for interpretation. Disruption scores were not correlated with tumor purity (Spearman R = − 0.082, n = 2,066) or variant read support (Spearman R = 0.050, n = 33 ATRT samples with read support data). To increase the stringency of our SV calls, we filtered our scored SVs to remove potential artifacts and excluded any SVs that shared identical breakpoints with another variant. This resulted in 107,614 remaining scored variants, which are included in the analyses presented in Figs. 2 and 3 and the corresponding Supplementary Figures.

### Recurrently disrupted regions

To find patterns in variant disruption across the genome, we performed dimensionality reduction on disruption scores across all variants and samples. The genome was divided into 1 Mb bins across all chromosomes, and within each bin, only the highest-disruption variant from each sample was considered. This results in two sample-by-bin matrices of maximum disruption scores, one using CORR and one using MSE. We used UMAP to reduce this data to two dimensions which resulted in various small clusters of samples that are separate from the rest. These clusters were samples that all had at least one variant with disruption scores past a cutoff in one bin compared to the rest of the genome. We qualitatively evaluated all variants that overlap each of the selected bins and excluded bins where the variant effects were not strong. We also excluded bins where the predicted chromatin contact map for the reference sequence did not match experimental contact maps due to low quality data, since we are less confident in predictions in these regions. The remaining five bins were considered RDRs and labeled based on their chromosome: chr3, chr7, chr19, chrX-a, and chrX-b. Note that these analyses were performed considering BNDs as individual variants and without excluding initial variants from progressive samples. While most progressive samples have different variants than initial ones, there is some overlap (Supplementary Table 3). Additionally, some SVs are repeated across multiple samples from the same individual, although this only occurs in the chr7 RDR.

### Comparing initial and progressive samples

To compare initial samples to progressive samples from the same individual, we took all individuals that had at least one initial and one progressive sample. If more than one sample of each category was present, we randomly chose one of the samples and made sure that we used that chosen sample in all downstream analyses. We then removed all SVs found in the progressive sample that were also present in the initial sample from the same individual, based on the SV coordinates and type. The following analyses were performed using the subsetted progressive sample SVs. To get log fold change in SV count (Fig. 1e), we used the total number of SVs in the initial versus progressive sample. To get t-test statistics and p-values (Fig. 2d), we performed a Welch’s t-test between scores from the initial and progressive sample.

### Defining disruptive variants and genes

To set a disruption score cutoff above which a variant is qualified as disruptive, we used a qualitative approach to select the minimum disruption score percentile above which SVs cause visual structural changes in the ~ 1 Mb window. To do so, we evaluated a range of cutoffs between the 90th to the 99th percentile, with 1% intervals, of both MSE and correlation. At each cutoff, the 10 lowest scoring variants above the cutoff were visualized, five based on MSE and five based on CORR. At the 98th percentile, few of the SVs caused structural differences, while at the 99th percentile, most did, suggesting that the optimal cutoff lies somewhere in between. We therefore next evaluated cutoffs between the 98.5th and 99.9th percentile, with 0.1% percent intervals. The 98.8th percentile was the lowest cutoff above which most SVs resulted in clear structural differences, so this was chosen as the disruption cutoff. Using this approach, 353 variants were labeled as disruptive across all samples and variant types. This subset mainly included DELs and did not include any INVs, which don’t cause as strong of structural changes. Of note, this cutoff does not signify that variants with lower scores are not structurally different but rather represents a stringent categorization of variants that are most likely to be disruptive. Genes within 300 kb of either end of each disrupted variant, but not overlapping the variant, were labeled as disrupted genes (n = 2,003).

### Epigenetic data processing

Raw ChIP-seq and ATAC-seq data from DIPG007, KNS42, D283 and BT16 cell lines (see Data Availability for accessions) were processed with the ENCODE data processing pipelines (https://github.com/ENCODE-DCC/chip-seq-pipeline2, https://github.com/ENCODE-DCC/atac-seq-pipeline) to produce .bam and peak files.

### ABC-modified scoring

We developed an ABC weighted disruption score, extending SuPreMo using ideas adapted from the ABC Model^25^. In brief, around 150,000 candidate regulatory elements (~ 500 bp long) were identified from H3K27ac ChIP-seq and ATAC-seq data from DIPG007, KNS42, D283 and BT16 cell lines. The Activity score from the ABC model was derived from the geometric mean of read counts of ATAC-seq and H3K27ac sites. Using Akita, a 1D genome disruption track was generated that represents the MSE between the REF and ALT maps for each of the 448 bins in a given prediction window. Any bin overlapping a 500-bp ABC enhancer is assigned the corresponding ABC Activity score; other bins have a score of 0. For non-BND SVs, the final disruption score is a weighted average of the 1D disruption track and the ABC Activity score track. For BNDs with multiple breakpoints, the final disruption score is the average of all weighted averages of the 1D disruption track and ABC activity track.

### Identifying upweighted variants from ABC-modified scoring

The ABC-modified scoring method was applied to the top 10% of disruptive variants from four tumor types. In order to identify variants that were upweighted, we ranked the variants based on their disruption score, for both the (1) original, unweighted MSE score and (2) ABC-weighted MSE score. We then calculated the difference in rank between the two scores, where a positive value indicates an increase in rank for the ABC-weighted score. The difference in rank was normalized based on the total number of variants in each dataset, and a normalized rank score $\ge$|40| was used to define upweighted and downweighted variants. This corresponded to < 1% (0.54–0.79%) of all variants across these four tumor types.

### Gene ontology analyses

Gene ontology (GO) analyses were performed with clusterProfiler in R. GO was performed using a background set of all genes within 300 kb of variants. For recurrently disrupted genes, the 249 genes within 300 kb of highly disruptive variants were used. For up- and down- weighted genes, genes in the 300 kb region upstream and downstream of upweighted variants from the ABC scoring method were used. For the latter, background genes were near SVs of the same tumor type.

## Supplementary Information

Supplementary Information 1.

Supplementary Information 2.

Supplementary Information 3.

Supplementary Information 4.

Supplementary Information 5.

Supplementary Information 6.

Supplementary Information 7.

## Data Availability

ChIP-seq for DIPG007 and KNS42 cell lines were obtained from the Gene Expression Omnibus with accession code GSE162976. ChIP-seq for D283 and BT16 were obtained from GSE92585 and GSE174446, respectively. ATAC-seq for D283, DIPG007, and KNS42 cell lines were obtained from GSE92585, GSE229453 and GSE162831, respectively. ATAC-seq for BT16 were obtained from ^45^ (https://paperpile.com/c/cgnLqO/yTPjQ). SuPremo-Akita scores and other results are available with this paper and its Supplementary Information.

## Code availability

The code to run SuPreMo-Akita was previously published and can be found at https://github.com/ketringjoni/SuPreMo.
